# Postoperative Quality of Life and Sexual Function in Premenopausal Women Undergoing Laparoscopic Myomectomy for Symptomatic Fibroids: A Prospective Observational Cohort Study

**DOI:** 10.1371/journal.pone.0166659

**Published:** 2016-11-29

**Authors:** Julia Caroline Radosa, Christoph Georg Radosa, Russalina Mavrova, Stefan Wagenpfeil, Amr Hamza, Ralf Joukhadar, Sascha Baum, Maria Karsten, Ingolf Juhasz-Boess, Erich-Franz Solomayer, Marc Philipp Radosa

**Affiliations:** 1 Department of Gynecology & Obstetrics, Saarland University Hospital, Homburg/Saar, Germany; 2 Department of Radiology, Dresden University Hospital, Dresden, Germany; 3 Institute of Medical Biometry, Epidemiology & Medical Informatics, Saarland University Hospital, Homburg/Saar, Germany; 4 Department of Gynecology, Charite Berlin, Berlin/Germany; 5 Department of Gynecology and Obstetrics, Jena University Hospital, Jena, Germany; Duke University, UNITED STATES

## Abstract

**Introduction:**

Uterine leiomyomas are the most common benign gynecologic tumors. To date laparoscopy myomectomy is the gold standard for treatment of symptomatic fibroids in reproductive-aged women. Detailed counseling about the effects of this procedure on postoperative sexuality and quality of life is important in these patients. However, available data on these subjects are limited and contradictory. The aim of this study was to assess sexual function and quality of life in premenopausal women undergoing laparoscopic myomectomy for symptomatic uterine fibroids.

**Material and Methods:**

All premenopausal women who underwent laparoscopic myomectomy for symptomatic fibroids between April 2012 and August 2014 at a tertiary university center were enrolled in this prospective observational cohort study. Sexual function and quality of life were assessed for the pre- and postoperative (six months post-operatively) state using two validated questionnaires, the Female Sexual Function Index (FSFI) and the European Quality of Life Five-Dimension Scale (EQ-5D).

**Results:**

Ninety-five of the 115 (83%) eligible patients completed the study. Overall a significant improvement in quality of life and sexual function was observed in the study cohort: Median FSFI (28 (18.7–35.2)) and EQ-5D scores (1 (0.61–1) after laparoscopic myomectomy were significantly higher than preoperative scores (21.2 (5.2–33.5); 0.9 (0.2–1); *p* ≤ 0.01). The number, position and localization of the largest fibroids were not correlated with pre- or postoperative sexual function or quality of life.

**Conclusion:**

Laparoscopic myomectomy might have positive short-term effects on postoperative quality of life and sexual function in premenopausal women suffering from symptomatic fibroids.

## Introduction

Uterine leiomyomas are the most common benign gynecologic tumors [1], with an 80% incidence [2, 3] and clinical appearance in approximately 25% of reproductive-aged women [4]. Symptoms (e.g. bleeding disorders, pelvic pressure/pain, reproductive dysfunction) [5, 6] correlate with fibroid number, size, position, localization and degenerative changes of fibroids [7] and can affect patients`quality of life and sexual function [8, 9]. Despite the recent introduction of pharmacological and radiologic interventional approaches, surgery (laparoscopic myomectomy or conventional myomectomy via laparotomy) remains one of the main treatment options of symptomatic fibroids in reproductive-aged women [6, 10]. Patient-reported parameters (i.e. postoperative quality of life and sexual function) may be used to assess treatment outcomes, supplementing traditionally used peri- and postoperative parameters (e.g. complication rates, blood loss, duration of surgery) which correlate insufficiently with treatment success in terms of symptom reduction [9, 11]. Few studies have evaluated patients-reported outcomes after surgical fibroid treatment. Myomectomy via laparotomy and hysteroscopic approach were found to improve quality of life in premenopausal women [9], but patient-reported outcomes differ between open and laparoscopic surgery and cannot be generalized to laparoscopic procedures [12, 13]. We conducted a comprehensive literature research, systematically searching MEDLINE (PubMed) using the keywords “laparoscopic myomectomy, quality of life and sexual function”. We found 29 articles, which matched the keywords, but none of these assessed postoperative quality of life and sexual function in a standardized way in a collective of exclusively premenopausal patients undergoing laparoscopic myomectomy. A few studies have evaluated patient-reported outcomes of symptomatic fibroid treatment performed using interventional radiology approaches (e.g., embolization, high-intensity ablation), but premenopausal patients who desire future pregnancy have been excluded in most cases due to limited experience with and long-term effects of these techniques, which limits the generalizability of these findings [14–16]. Our study aimed to address this critical niche by evaluating postoperative quality of life and sexual function in a valid and reproducible manner using the Female Sexual Function Index (FSFI) and European Quality of Life Five-Dimension Scale (EQ-5D) in premenopausal women with symptomatic fibroids undergoing laparoscopic myomectomy.

## Material and Methods

All patients who underwent laparoscopic myomectomy between April 2012 and August 2014 at the Department of Gynecology and Obstetrics at a tertiary university center were enrolled in this prospective observational cohort study. All participants provided written informed consent and ethical approval from the Saarland institutional review board was obtained for this study (registration number 180/13). The trial was registered in the German Clinical Trials Register (DRKS) (trial registration number DRKS00005622). The following inclusion criteria were applied: 1) premenopausal status at time of surgery, assessed according to World Health Organization criteria [17]; 2) laparoscopic myomectomy performed without concurrent unilateral or bilateral adnexectomy; and 3) American Society of Anesthesiologists physical status classification of I or II, assessed preoperatively by Anesthesiology. Exclusion criteria were: 1) refusal to participate in the study; 2) prior treatment with gonadotropin-releasing hormone agonists or ulipristal acetate or other perioperative hormonal treatment; 3) current endometriosis or history of surgery to treat endometriosis 4) intra-operatively diagnosed adnexal pathology requiring uni- or bilateral oophorectomy. Preoperatively, all patients provided medical history and underwent gynecologic examination and transvaginal ultrasound (Philips CX 50, Philips, Hamburg/Germany) to document the size, localization and number of fibroids. Basic laboratory tests were performed upon admission. As described in detail previously, all surgeries were performed in the hospital`s Department of Gynecology under general anesthesia by an experienced consultant or registrar [11, 18]. All patients received perioperative antibiotics (single-shot cefuroxime (1.5 g i.v.; Fresenius, Bad Homburg/Germany) and metronidazole (0.5 g i.v; Fresenius, Bad Homburg/Germany) and low-molecular-weight heparin (enoxaparin 40 mg, s.c. once a day; Sanofi-Aventis, Paris/France) as thromboembolism prophylaxis and indwelling catheter were used until the first postoperative morning. Laparoscopic myomectomy was performed in the lithotomy position using four ports as described previously [18]: one 10-mm optic trocar inserted through the umbilicus; two 5-mm working trocars inserted through the infero-lateral abdominal wall, two fingers’ distance above the iliac crest; and one 15-mm working trocar in the suprapubic area, two fingers`distance above the symphysis pubis. The laparoscopic technique used in this study has been described in detail elsewhere [18, 19]. A diluted (1:200.000) epinephrine solution (Xylonest, Astra-Zeneca, Wedel/Germany) was injected into the serosa and the myometrium overlying the fibroid for vasoconstriction. After vertical incision of the serosa and myometrium, enucleation was performed. The myometrial defect was closed with intracorporal interrupted single- or two-layer sutures (Vicryl 1; Ethicon, Noderstedt/Germany). Fibroids were morcellated with an electric morcellator (Rotocut G1; Storz, Tuttlingen/Germany) and extracted through the medial trocar. The duration of surgery (from umbilical cutaneous incision to closure of all trocar sites), number, position and localization of fibroids, classified according to the International Federation of Gynecology and Obstetris (FIGO) system for uterine leiomyomas [20], intraoperative occurrence of adverse events (defined as iatrogenic injury of the bladder, bowel, ureter, or any major vessel; need for intraoperative conversion to laparotomy or abandonment of the intended surgical procedure), patients`comorbidities, daily medication and history and number of previous operations (using the previous surgery score as described by van Evert et al.: calculated as one point per laparoscopy plus two points per laparotomy (including cesarean sections)) [21], pre- and postoperative hemoglobin concentrations, duration of postoperative hospitalization, first postoperative day to day of discharge, and surgical complications, according to the Clavien-Dindo Classification [22] were documented.

Patients were advised to avoid sexual intercourse for six weeks after laparoscopic myomectomy and to avoid pregnancy for six months postoperatively. In cases of opening of the uterine cavity patients were counseled to undergo a primary cesarean section in case of pregnancy in order to minimize the risk for uterine rupture. Main outcome measures were patients`preoperative and six-month postoperative sexual function and quality of life. Enrollment, signing of the consent from, delivery and return of the preoperative questionnaire took place at the first preoperative visit of the patients at our outpatient department. The timeline between this first visit and the date of surgery ranged between three to eight weeks. The postoperative questionnaires were sent to the patients by postal mail six months after the operation. The patients returned the questionnaires by postal mail within one to six weeks. In order to ensure confidentiality a study number was assigned to all patients upon registration.

Sexual function and quality of life were assessed using FSFI and EQ-5D. As described previously, the FSFI is a validated, multidimensional, self-reported instrument for the assessment of female sexual function [11, 23, 24]. It consists of 19 items evaluating the FSFI lubrication, sexual arousal, orgasm, sexual satisfaction, and pain. The total score (range: 2 (severe sexual dysfunction) to 36 (full sexual function)) is calculated by summing the five domain score and interpreted by comparison with age- and population-dependent reference values for normal and impaired sexual function [25] [24]. A FSFI score ≤ 26.55 is considered to indicate sexual dysfunction [25]. The EQ-5D is a standardized, validated instrument used to measure an individual’s health status [11, 26]. The questionnaire consists of two parts; the descriptive portion assesses five dimensions of health-related quality of life (mobility, self-care, usual activities, pain/discomfort, and anxiety/depression) using three levels (no problem, some/moderate problems, and extreme problems). Using the time trade-off method, an age- and ethnicity-specific EQ-5D index score (EQ-5D score; range: 1 (full quality of life) to—0.207 (worst health status)) was calculated for each patient. The second part of the questionnaire comprises a vertical visual analog scale (VAS) ranging from optimal health status (100 points) to worst health status (0 points). Differences in pre- and postoperative FSFI, EQ-5D and VAS scores (FSFI-d, EQ-5D-d and VAS-d respectively) were determined.

A sample size of at least 55 patients was sought to achieve 80% power to detect a mean paired difference of 2.0 score points on FSFI changes between baseline and postoperative values with an estimated 5.0 standard deviation of differences and a significance level (alpha) of 0.05 using a two-sided Wilcoxon test. We inflated the sample size to 115 patients to allow for drop-outs and incomplete questionnaires. We used descriptive statistics and the Kolmogorov–Smirnov test to assess normality of FSFI, EQ-5D and VAS score distributions. As the data was not normally distributed, we used the Wilcoxon signed-rank test for dependent samples to compare pre- and postoperative values and the Mann–Whitney *U*-test to assess differences between groups with FSFI scores ≤ 26.55 and >26.45 and differences between FSFI, EQ-5D and VAS values by main indication for myomectomy. Spearman`s rank correlation coefficient was used to assess correlations between the position, localization and number of fibroids and FSFI, EQ-5D and VAS scores. For pre- and postoperative comparison of patients living in a relationship McNemar`s exact test was used. Data are reported as means ± standard deviations, or as medians (ranges) for variables with non-Gaussian distribution. Data were collected in an Excel database (Excel 2010; Microsoft Corporation, Redmond, WA/USA), statistical calculations were performed with SPSS software (Version 19; SPSS Inc., Chicago, IL, USA) and PASS (Version 12.0.2.; Kaysville, UT/USA) was used for sample size calculation.

## Results

**Fig 1:**
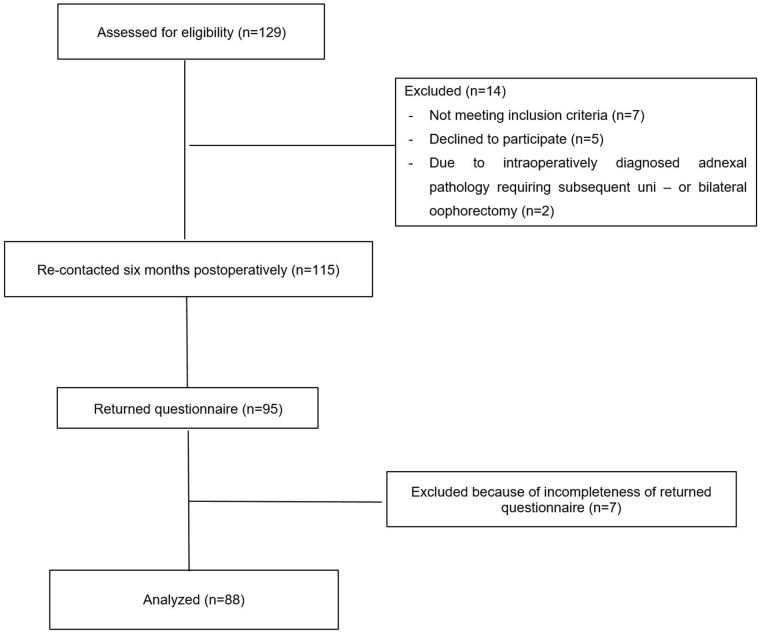
Flow chart of study design (n = 88).

During the study period a total of 129 patients underwent laparoscopic myomectomy. Fourteen patients were excluded; seven did not meet the inclusion criteria, five declined to participate in the study and two patients were excluded post-operatively due to intra-operatively diagnosed adnexal pathology requiring subsequent uni- or bilateral oophorectomy. Ninety-five of 115 study participants, returned questionnaires post-operatively (83% response rate). Seven patients were excluded due to incomplete questionnaire responses. Thus, the analyses included a total of 88 premenopausal patients who underwent laparoscopic myomectomy (Fig 1).

Mean age and BMI at time of operation were 35.4 years (± 5.5) and 25.4 kg/m^2^ (± 6.4).

66 patients (75%) stated that they had never undergone surgery before and the 22 patients (25%) who had undergone surgery before had a median previous surgery score of one (range 1–3). 18 patients (20%) had a history of musculoskeletal (seven patients), endocrinologic (five patients), cardiovascular (two patients), auto-immune (two patients) or ophthalmologic (two patients) comorbidities and seven patients were on daily medication for these diseases. These patients`characteristics were not correlated with pre- or postoperative sexual function or quality of life.

Main indications for surgery were lower abdominal pain (47.7%), infertility/sterility (30.7%), abnormal uterine bleeding (14.8%), preoperative symptomatic pelvic mass (4.5%) and other benign pathologies (2.3%). The mean duration of surgery was 121.5 (± 63.2) minutes and the mean reduction in hemoglobin was 0.6 (± 0.5) g/dl. A mean number of 2.3 (± 2.1) fibroids with an average maximum diameter of 52.8 (± 21.8) mm were removed (Table 1).

**Table 1:** Patients`characteristics and surgical outcomes (n = 88).

	**Mean**	**SD**
Age (years)	35.4	5.5
BMI (kg/m^2^)	25.4	6.4
Duration of surgery (minutes)	121.5	63.2
Hemoglobin drop (g/dl)	0.6	0.5
Postoperative hospitalization (days)	3.4	1.4
Average diameter of leading fibroid (mm)	52.8	21.8
Number of fibroids removed	2.3	2.1
	**n**	**%**
***Main indication for myomectomy***		
Lower abdominal pain	42	47.7
Infertility/Sterility related to myoma	27	30.7
Abnormal uterine bleeding	13	14.8
Preoperative symptomatic pelvic mass	4	4.5
Other	2	2.3
***Number of myomas***		
< 3	61	69.3
≥ 3	27	30.7
Localization of largest fibroid*		
Submucosal with intramural component (FIGO SM-2)	6	6.8
Intramural (FIGO O-3/O-4)	44	50
Subserous (FIGO O-5/O-6)	31	35.2
Subserous pedunculated (FIGO O-7)	5	5.7
Broad and/or round ligament with no relation to myometrium (FIGO O-8)	2	2.3
***Position of largest fibroid***		
Anterior	23	26.1
Posterior	31	35.2
Lateral	11	12.6
Fundal	23	26.1
Postoperative complications (Clavien-Dindo)**		
Grad I-II	4	4.6
Grad III	1	1.1
Grad IV-V	-	-

*Classification according to the FIGO classification system of uterine leimyomas (16)

** Classified according to the Clavien-Dindo classification for surgical complications (17)

Sixty-one (69.3%) patients had one or two myomas, 27 (30.7%) patients had three or more myomas. Regarding the localization of the largest fibroids, 44 (50%) were intramural (FIGO O-3/O-4), 31 (35.2%) were subserous (FIGO O-5/O-6), 6 (6.8%) were submucosal (FIGO SM-2), 5 (5.7%) were subserous pedunculated (FIGO O-7) and 2 (2.3%) were broad or round ligament fibroids with no relation to the myometrium (FIGO O-8). The rates of posterior, anterior, fundal and lateral fibroid position were 35.2% (n = 31), 26.1% (n = 23), 26.1% (n = 23) and 12.6% (n = 11) respectively (Table 1). Preoperative diagnosis of uterine fibroids was confirmed by postoperative histopathologic examination in all cases. These examinations revealed no malignant lesion within the study cohort. In two patients, the initial laparoscopic route was abandoned and myomectomy was performed by laparotomy due to the presence of extensive adhesions (n = 1) or multiple intramural fibroids (n = 1). In the postoperative course a total of five complications (5.7% complication rate) were noted. In four patients, these complications were classified as minor (grade I (n = 3) or grade II (n = 1)) complications according to the Clavien-Dindo system. One major (grade III) complication (requirement for surgical intervention due to postoperative intraabdominal bleeding) was recorded.

The percentages of women living in a relationship did not differ significantly between the pre- (86.4%) and postoperative (88.4%) status (*p* = 1). Median preoperative FSFI, EQ-5D and VAS scores were 21.1 (5.2–33.5), 0.9 (0.2–1) and 70 (15–100) respectively. Median postoperative scores 28 (18.7–35.2), 1 (0.6–1) and 90 (50–100) respectively, were significantly higher than preoperative scores (*p*<0.001; Table 2).

**Table 2:** Comparisons of pre- and postoperative FSFI, EQ-5D, VAS scores and FSFI. domains (n = 88).

	Preoperative		Postoperative	
	Median	Range	Median	Range
***Main scores***				
FSFI	21.1	5.2–33.5	28*	18.7–35.2
EQ-5D	0.9	0.2–1	1*	0.6–1
VAS	70	15–100	90*	50–100
***FSFI domains***				
Desire	3	0.6–6	4*	1.2–6
Arousal	3.2	0–5.7	4.5*	1.2–6
Lubrication	4	0.6–6	5*	0.9–6
Orgasmn	3.6	0–6	4.4*	1.2–6
Satisfaction	3.4	0.4–6	4.8*	1.2–6
Pain	4.6	0–6	6*	2–6

* p<0.001 for comparison of pre- and postoperative FSFI, EQ-5D, VAS scores and FSFI domains, Wilcoxon signed-rank test for dependent samples was used to compare pre- and postoperative values

Postoperative FSFI domain scores (desire, lubrication, arousal, orgasm, satisfaction, pain) were also significantly higher than preoperative scores (*p*<0.001; Table 2). Patients with normal preoperative FSFI scores (≥ 26.55; n = 16) [27] showed no significant increase in postoperative FSFI (*p* = 0.45), EQ-5D (*p* = 0.09) or VAS score (*p* = 0.55). Regarding the main indication for myomectomy, patients treated for lower abdominal pain showed the lowest preoperative FSFI scores and the highest increase in FSFI-values compared to patients treated for other indications (16.6 (5.2–20.5) vs. 24.7 (20.6–33.5) p<0.001; 8.2 (1–23.2) vs. 4.7 (0–10.6) p<0.001. With median preoperative FSFI scores of 23.1 (21.6–23.7) and 25.4 (20.6–30.6) patients treated for abnormal uterine bleeding and infertility also showed an impaired preoperative sexual function (FSFI < 26.55). Both groups had a significant increase in postoperative FSFI scores (28.9 (24.5–33.6) p<0.001; 29.9 (25–35.2) p<0.001). Looking at EQ-5D and VAS values there were no significant differences between the five indication groups.

FSFI, EQ-5D and VAS scores were not correlated with the position or localization of the largest fibroid or the total number of fibroids (*p*>0.05). Regarding the differences between postoperative and preoperative scores, subserous pedunculated (FIGO O-7) myomas showed significant lower FSFI- (FSFI-d) differences and significantly lower differences in the FSFI subdomain pain (pain-d) when compared with other localizations and subserous (FIGO O-5/O-6) myomas were associated with significantly higher values in these two categories (p<0.05; Table 3).

**Table 3:** Correlation of pain- and FSFI-score differences with number, localization and position of fibroid (n = 88).

	Pain-d			FSFI-d		
	Median	Range	*p*	Median	Range	*p*
***Number of myomas***						
< 3	1.2	0–6	0.1	5.6	0–23	0.3
≥ 3	1.2	0–3.2	0.3	4.9	0–20	0.9
***Localization of largest fibroid***						
Submucosal with intramuralcomponent (FIGO SM-2)	4.9	1–8	0.4	1.2	0–2.8	0.5
Intramural (FIGO O-3/O-4)	6.5	1–23	0.3	1.6	0–5.6	0.4
Subserous (FIGO O-5/O-6)	4.5	0–14	**< 0.05**	1.2	0–6	**0.04**
Subserous pedunculated(FIGO O-7)	14.5	5–20	**0.01**	3.2	1.2–6	**0.01**
Broad and/or round ligamentwith no relation tomyometrium (FIGO O-8)	7.9	5–11	0.7	1.2	0.8–1.6	0.74
***Position of largest fibroid***						
Anterior	6.4	1–20	0.5	1.4	0.8–6	0.7
Posterior	5.2	0–19	0.7	1.2	0–6	0.6
Lateral	6.2	1–17	0.7	1.2	0–5.6	0.2
Fundal	5.1	0–23	0.5	0.8	0–3.2	0.5

-d: differences between postoperative and preoperative pain- and FSFI-scores; Spearman`s rank correlation coefficient was used to assess correlations between the position, localization and number of fibroids and score differences

Patients in whom conversion to laparotomy was performed (n = 2) or a major postoperative complication was noted (n = 1), all (n = 3) completed the questionnaires post-operatively and postoperative FSFI, EQ-5D, and VAS scores did not differ significantly from those of the rest of the cohort.

## Discussion

This study was designed to evaluate quality of life and sexual function following laparoscopic myomectomy in a valid and reproducible manner. To address this methodological challenge, we assessed the impacts of this treatment in a representative cohort of premenopausal patients with symptomatic fibroids and predominantly impaired preoperative sexual function presenting at a tertiary university center. Laparoscopic myomectomy seemed to have positive short-term effects on postoperative quality of life and sexual function in this cohort. In a subgroup analysis we found patients with impaired preoperative sexual function to benefit most from the procedure.

Two recent studies have used similar designs to evaluate postoperative quality of life and sexual function in patients undergoing abdominal myomectomy for symptomatic fibroids. Dilek and colleagues evaluated the impact of myomectomy via laparotomy on women`s quality of life using the Short Form Health Survey and reported postoperative improvement in “pain” “vitality” and “general health” but no improvement in “physical function” or “mental health” scores [9]. Similarly Ertunc and colleagues assessed the effect of myomectomy via laparotomy on patients‘ sexual function using the FSFI score [8]. They found significant increases in postoperative total FSFI and in the FSFI subdomain pain scores relative to preoperative scores. Our findings are in partial agreement with the results of these studies. However we found significant increases in all quality of life aspects and all FSFI subdomains. These discrepancies may be related to differences in surgical approach. In comparison with laparoscopic surgery, open surgery is associated with higher postoperative wound pain levels and increased time until resumption of normal activities of daily life [28]. Some authors have suggested that higher postoperative wound pain levels and adhesion formation result in long-term impairment of quality of life after laparotomic procedures [29]. Griffin and colleagues observed lower postoperative wound pain levels and an accelerated return to work in women who underwent laparoscopic-robotic myomectomy compared with those who underwent myomectomy via laparotomy [28]. In addition to the clinical superiority of this surgical technique, our findings indicate that minimal-invasive myomectomy may also improve patient-reported outcomes relative to open myomectomy.

Previous studies have shown that fibroid-related symptoms and impaired quality of life and sexual function are not correlated with the number, position or localization of fibroids [30]. We found greater improvement in sexual function among women with subserous and less improvement in those with subserous pedunculated myomas. These findings may not be directly related to fibroid localization, but may be a result of differences in postoperative symptom relief, as reflected by FSFI subdomain pain-differences values. These values were greater in patients with subserous and smaller in those with subserous pedunculated fibroids.

Several studies have provided support for the hypothesis that improved post-myomectomy quality of life and sexual function are derived mostly from symptom relief achieved by the surgery [31]. Wang and colleagues demonstrated that myomectomy normalized quality of life by improving symptoms in patients with symptomatic fibroids [32]. This assumption is also supported by the lack of significant changes in FSFI and EQ-5D scores in patients with normal preoperative FSFI scores in the present study. The therapeutic effect achieved by myomectomy seems to manifest only in patients with impaired preoperative sexual function and might originate in somatic symptom relief. Correspondingly we found the highest impairment in sexual function preoperatively and the highest effect of myomectomy in patients undergoing surgery for abdominal pain as main indication. However patients undergoing laparoscopic myomectomy for uterine bleeding disorders and infertility also presented with impaired preoperative sexual function and obtained significant improvement in sexual function by myomectomy.

According to the recent U. S. Food and Drug Administration safety warning about the use of laparoscopic power morcellation, laparoscopic myomectomy should be conducted only after careful benefit-risk evaluation and in patients for whom it is considered the best therapeutic option [33]. In this context preoperative evaluation using the FSFI and EQ-5D might be of value in predicting treatment success and selecting patients most likely to benefit from this treatment.

This study showed that laparoscopic myomectomy in premenopausal patients with symptomatic fibroids might significantly affect patient-reported outcomes within a short-term follow-up period. Several authors have documented increased sexual function and quality of life following surgical treatment of benign uterine pathologies, but these differences were much smaller than observed in this study [8, 11]. A possible explanation for these differences in findings could be the fact that we evaluated quality of life and sexual function six months after laparoscopic myomectomy. These short-term results cannot be generalized to mid- and long-term outcomes. Fibroid recurrence and persistent postoperative infertility can affect quality of life and sexual function in the long term, thereby; altering the long-term outcomes of initially successful treatment [19, 34]. We did not assess fibroid recurrence or fertility rates in this study because valid information could not be obtained within the follow-up period. Further investigations are needed to evaluate the long-term effects of laparoscopic myomectomy on patient-reported outcomes.

In a minor, yet considerable percentage, (3.4%) of patients in our study the postoperative course was altered substantially by intraoperative conversion to laparotomy or the occurrence of major postoperative complications. Although these patients’ postoperative FSFI, EQ-5D and VAS scores did not differ from those of the rest of the study population, adverse events related to laparoscopic fibroid treatment might have considerable negative impacts on postoperative quality of life and sexual function in individual cases. This factor should be considered in the preoperative counseling of patients suffering from symptomatic fibroids.

A limitation of the study is the use of an open-series design with no control group. Spies and colleagues noted significant improvement in the quality of life of patients with symptomatic fibroids following radiologic interventional or surgical (predominantly via laparotomy) treatment, in contrast to the lack of positive changes during the study period an untreated control group [35]. In light of these findings, we found it ethically questionable to assign a portion of patients presenting at our hospital for the treatment of symptomatic fibroids to a non-interventional control group for the purposes of this study. Due to this methodological challenge the possible contributions of additional factors other than the ones assessed to the observed postoperative changes in patient-reported outcomes cannot be excluded.

This study does not provide a comparison between laparoscopic and open myomectomy and to the best of our knowledge there is no currently available data comparing these surgical approaches in terms of sexual function and quality of life. In comparison to the two studies cited before, which assessed sexual function and quality of life after open myomectomy, patients in our cohort showed better outcomes (8, 9). This result might indicate an advantage for the minimal-invasive approach over open myomectomy in terms of these patient-reported outcomes. However further research will be needed in order to sufficiently answer this question. Patients’ preoperative sexual function and quality of life were assessed in close temporal relation to the decision to undergo surgical treatment and to the date of surgery itself. This constitutes a potential limitation with regards to the external validity of our data, since the preoperative period is loaded with emotional issues that might interfere “per se” on quality of life and sexual function. Assessment of quality of life and sexual function in our patients may have been affected by patients’ potential anxiety originating from forthcoming intervention and on top of that psychopathologic issues cannot be excluded. In our study sample, we did not observe a significant change in pre- and postoperative values for the EQ5-D subcategory anxiety/depression, indicating a potential systematic bias due to patients’ anxiety caused by the impending surgery. However, we acknowledge, that such preoperative anxiety could have affected preoperative FSFI and EQ5-D values in some of our study patients.

Another limitation might be constituted by the special nature of our cohort. Almost one third of our patients underwent laparoscopic myomectomy with “infertility” as main indication, which is a relative weak indication and on the contrary, only 15% underwent laparoscopic myomectomy for abnormal uterine bleeding/menorrhagia, which is instead considered one of the major indications to fibroid surgery. In our opinion a possible explanations for this “untypical” distribution of fibroid-related symptoms could be that our study collective consisted exclusively of premenopausal patients with a mean age of 35.4 years. In this premenopausal cohort indications for myomectomy and presenting symptoms might be different compared to peri- or postmenopausal cohorts as described in other publications [36].

Impaired sexual function is generally not regarded as an indication for surgical treatment of otherwise asymptomatic uterine fibroids. However, given the significant postoperative improvement in sexual function observed in the majority of study participants, the question whether poor sexual function should be included as an indication for myomectomy should be addressed in future research.

In summary we found that premenopausal women undergoing laparoscopic myomectomy for symptomatic fibroids might have statistical significant short-term improvements in quality of life and sexual function. Patients with impaired preoperative sexual function seemed to benefit most from surgery. These findings might help to improve selecting patients for laparoscopic myomectomy. However long-term outcomes of laparoscopic myomectomy have to be further evaluated.

## Supporting Information

S1 File Strobe checklist completed. (DOCX)

S2 File Trial study protocol German. (DOC)

S3 File Trial study protocol English. (DOC)
