# Supplementary material for: Postoperative Quality of Life and Sexual Function in Premenopausal Women Undergoing Laparoscopic Myomectomy for Symptomatic Fibroids: A Prospective Observational Cohort Study
Source: PLoS One. 2016 Nov 29;11(11):e0166659. doi: 10.1371/journal.pone.0166659 (PMC5127523; doi:10.1371/journal.pone.0166659)
Supplement: S2 File — (DOC) [file pone.0166659.s002.doc]

Antrag an die Ethikkommission der Ärztekammer des Saarlandes zur Beurteilung eines medizinschen Forschungsvorhabens im Fachbereich Medizin der Universität des Saarlandes

**1. Formales**

1.1 Bezeichnung des Vorhabens

Befragung von Patientinnen zum Thema Lebensqualität und Sexualität nach gynäkologischen Operationen mittels eines standardisiereten Fragebogens

1.2 Name des verantwortlichen Leiters und seiner Mitbetreuenden Ärzte

Verantwortlicher Leiter:

Prof. Dr. med. E.-F. Solomayer

(Klinikdirektor der Klinik für Gynäkologie und Geburtshilfe der Universitätsklinik Homburg

Dr. med. S. Baum (Leitender Oberarzt Universitätsfrauenklinik Homburg)

Dr. med. J. Radosa (Assistenzärztin Universitätsfrauenklinik Homburg)

Mitbetreuende Ärzte:

C. Kastl (Assistenzärztin Universitätsfrauenklinik Homburg)

# 1.2.1 Wissenschaftlicher Werdegang des Projektleiters Prof. Dr. med. E.-F. Solomayer

1986-1990 Studium der Humanmedizin in Klausenburg

1991-1995 Studium der Humanmedizin an der Ruprecht-Karls Universität Heidelberg

1995-2001 AiP und wissenschaftlicher Assistent an der Universitäts-Frauenklinik Heidelberg

11/2000 Anerkennung als Facharzt für Gynäkologie und Geburtshilfe

04/2001 Oberarzt der Universitäts-Frauenklinik Tübingen

Leitung des Forschungslabors

11/2004 Geschäftsführender Oberarzt

01/2005 Fakultative Weiterbildung „Operative Gynäkologie“

02/2005 Fakultative Weiterbildung „Geburtshilfe und Perinatologie“

06/2005 Leitender Oberarzt

07/2005 Stellv. ärztliche Leitung der Hebammenschule

09/2005 Stellv. Ärztlicher Direktor

03/2006 Ausserplanmäßiger Professor

10/2009 Berufung auf den Lehrstuhl für Gynäkologie, Geburtshilfe und Reproduktions- medizin an der Universität des Saarlandes in Homburg/Saa

1.3 Art und Anzahl der Prüfstellen

Das Vorhaben wird durchgeführt in der: Klinik für Frauenheilkunde, Geburtshilfe und

Reproduktionsmedizin

Klinikdirektor: Prof. Dr. med. E.-F. Solomayer

Kirrbergerstr. 100

66421 Homburg/Saar

Sonstige Prüfzentren: Entfällt

1.4 Name und Anschrift des Sponsors

Klinik für Frauenheilkunde, Geburtshilfe und Reproduktionsmedizin

Klinikdirektor: Prof. Dr. med. E.-F. Solomayer

Kirrbergerstr. 100

66421 Homburg/Saar

1.5 Wurde schon bei einer anderen Ethik-Kommission ein diesbezüglicher

Antrag gestellt?

Nein

1.5.1

Entfällt

1.5.2

Entfällt

**2. Beschreibung und wissenschaftliche Begründung des Projektes**

**2.1 Erläuterung des Studienziels**

Ziel ist die Gewinnung von Daten zur Lebensqualität, Entscheidungsfindung und Sexualität nach gynäkologischen Operationen. Dies soll mithilfe eines standardisierten, anonymen Fragebogens erfolgen, welcher an Patientinnen, die in der Universitätsfrauenklinik Homburg im Zeitraum ab 2008 operiert wurden, prä- und postoperativ versandt wird. Im Besonderen der Einfluss verschiedener Hysterektomieverfahren (vaginale Hysterektomie, totale laparoskopische Hysterektomie und suprazervikale Hysterektomie) auf die postoperative Lebensqualität und Sexualität soll untersucht werden. Dazu werden erstmalig zwei validierte Score Systeme zur Erhebung der Lebensqualität (EQ5D Score) und der Sexualität (FSFI – Female Sexual Function Index) verwendet.

2.2. Darstellung des bisherigen Wissenstandes

Bisher liegen kaum Studien zum Thema Lebensqualität und Sexualität nach Hysterektomie vor. Bei den ersten Untersuchungen zu diesem Thema handelt es sich um Studien mit kleinen Fallzahlen aus den 80er Jahren, welche oft empirische Daten oder Expertenmeinungen wiederspiegeln. Grundtonus der Arbeiten ist, dass es nach Hysterektomie zu einer Verschlechterung der Lebensqualität und Sexualität kommt (1).

Die ersten systematischen Arbeiten zu dem Thema stammen von der Arbeitsgruppe um Zobbe et al., welche 2004 eine Studie zum Vergleich der totalen laparoskopischen Hysterektomie und der suprazervikalen Hysterektomie veröffentlichten (2). Sie konnten zeigen, dass beide Hysterektomieverfahren zu einer Verbesserung der postoperativen Lebensqualität und zu keiner Verschlechterung der postoperativen Sexualität führten. Allerdings sind die Ergebnisse nur schwer objektivierbar da sowohl für die Lebensqualität als auch für die Sexualität keine standardisierten, validierten Erhebungsfragebögen verwendet wurden.

2.3 Vorgesehene Gesamtdauer aller Untersuchungen

Die Studie soll nach Vorliegen eines positiven Ethikvotums beginnen und wird voraussichtlich für zwei Jahre durchgeführt werden.

2.4 Begründung der Notwendigkeit der Studien an Menschen

Zur Erlangung der Daten ist die Befragung von Menschen unerlässlich

**2.4.1 Studie an gesunden Probanden?**

Entfällt

**2.4.2 Studie an Patienten?**

Entfällt

**2.4.3 Einschlusskriterien**

Eingeschlossen werden Patientinnen, welche sich an der Universitätsfrauenklinik Homburg aufgrund von gynäkologischen Erkankungen einer Operation unterziehen. Bevorzugt sollen zunächst Patientinnen eingeschlossen werden, welche sich aufgrund benigner uteriner Pathologien einer Hysterektomie unterzogen haben.

**2.4.4 Ausschlusskriterien**

Entfällt

2.4.5 Abbruchkriterien

Entfällt

**3. Literaturangaben**

1) Raboch et al.: Sex life following hysterectomy. Geburtshilfe

Frauenheilkd. 1985 Jan;45(1):48-5a

2) [Zobbe V](http://www.ncbi.nlm.nih.gov/pubmed?term=Zobbe V%5BAuthor%5D&cauthor=true&cauthor_uid=14756739) et al.: [Acta Obstet Gynecol Scand.](http://www.ncbi.nlm.nih.gov/pubmed/14756739#%23) 2004 Feb;83(2):191-6. Sexuality after total vs.

subtotal hysterectomy.

**4. Darlegung der vorraussichtlichen Vorteile und der Bedeutung der Studie für den Menschen**

**a) In der Heilkunde**

Durch Gewinnung genauere Daten zur postoperativen Sexualität und Lebensqualität nach Hysterektomie und dem Einfluss der verschiedenen Operationsverfahren auf das postoperative Outcome wäre eine bessere Beratung der Patientinnen präoperativ möglich.

**b) In der Forschung**

Entfällt

**4.1. Minderjährige Studienteilnehmer**

Entfällt

**4.2. Individuelle Indikation**

Entfällt

**5. Güterabwägung zwischen den Nachteilen und Risiken einerseits und dem**

**voraussichtlichen Nutzen andererseits**

**5.1.**

Durch die Teilnahme an der Studie entstehen den Patientinnen keinerlei Nachteile. Alle gewonnenen Daten werden nur in anonymisierter Form und streng vertraulich verwendet.

**5.2.**

Entfällt

**5.3**

Entfällt

**6. Angaben über den Inhalt der Aufklärungsgespräche mit den Studienteilnehmern**

**6.1**

Die Aufklärung über die Studienteilnahme erfolgt in Form eines schriftlichen Anschreibens, welches mit dem Fragebogen versendet wird (siehe Anhang). Die Rücksendung des Fragebogens ist freiwillig und in anonymisierter Form.

**6.2 Aufklärung über Widerruflichkeit der Einwilligung**

Die Einwilligung jederzeit ohne Angaben von Gründen und ohne Nachteile widerrufen werden kann.

**6.3**

Entfällt

**6.4.**

Entfällt

**6.5.**

Entfällt

**6.6**

Entfällt

**6.7**

Entfällt

**7. Beifügung eines Musters des verwendeten Einwilligungsformulars**

Siehe Anhang

**8. Nachweis einer ausreichenden Probandenversicherung**

Die Sammlung der Daten erfolgt im Rahmen medizinisch notwendigen Eingriffen, so dass der Versicherungsschutz über die übliche ärztliche Haftung ausreichend ist.

**9. Darlegung der Erfüllung etwaiger sonstiger Voraussetzungen für die Durch-**

**führung Studie**

Entfällt

**10. Information des Hausarztes**

Entfällt

**11.**

Entfällt
