# Supplementary material for: Postoperative Quality of Life and Sexual Function in Premenopausal Women Undergoing Laparoscopic Myomectomy for Symptomatic Fibroids: A Prospective Observational Cohort Study
Source: PLoS One. 2016 Nov 29;11(11):e0166659. doi: 10.1371/journal.pone.0166659 (PMC5127523; doi:10.1371/journal.pone.0166659)
Supplement: S3 File — (DOC) [file pone.0166659.s003.doc]

**Proposal to the Saarland ethical review board upon realisation of a medical study**

**1. Formal application**

1.1 Aim of the study

The aim of the study is the acquisition of information about postoperative sexual function and quality of life in patients undergoing surgery for benign gynecological pathologies

1.2 Principal investigators

Principal investigator

Prof. Dr. med. E.-F. Solomayer

(Head of department, Deparment of Gynecology and Obstetrics, Saarland university hospital)

Co-Investigators

Dr. med. S. Baum

(Attending, Deparment of Gynecology and Obstetrics, Saarland university hospital)

Dr. med. J. Radosa

(Fellow, Deparment of Gynecology and Obstetrics, Saarland university hospital))

C. Kastl (Fellow, Deparment of Gynecology and Obstetrics, Saarland university hospital))

# 1.2.1 Curriculum Vitae of principal investigator

1986-1990 Medical school of Clausenburg

1991-1995 Medical school, Heidelberg university

1995-2001 Fellow Department of Gynecology and Obstetrics, Heidelberg University

11/2000 Board certification for Gynecology and Obstetrics

04/2001 Attending, Department of Gynecology and Obstetrics, Tübingen University

hospital

11/2004 Attending in charge

01/2005 Board certification for surgical Gynecology

02/2005 Board certification for Obstetrics

10/2009 Head of department, Department of Gynecology and Obstetrics, Saarland University hospital

1.3 Number and specification of study hospital

The study will be conducted at the following institution:

Deparment of Gynecology and Obstetrics, Saarland university hospital

Kirrbergerstr. 100

66421 Homburg/Saar

Germany

Other institutions: None

1.4 Name and address of the sponsor

Deparment of Gynecology and Obstetrics, Saarland university hospital

Head of department: Professor Dr. med. E.-F. Solomayer (MD)

Kirrbergerstr. 100

66421 Homburg/Saar

Germany

1.5 Has there been another proposal to another ethical review board?

No

1.5.1

Not applicable

1.5.2

Not applicable

**2. Description of study design**

**2.1 Description of the aim of the study**

The aim of this study is the collection of data about the influence on postoperative sexual function and quality of life in patients` undergoing surgery for benign gynecologic pathologies. All women who underwent/undergo surgery (hysterectomy, myomectomy, Sacro-Kolpopexie, etc.) for benign gynecologic disease from 2008 onwards will be included. Sexual function and quality of life are assessed using two validated questionnaires, the Female Sexual Function Index (FSFI) and the European Quality of Life Five-Dimension Scale (EQ-5D). These values are assessed pre- and postoperatively.

2.2. Current scientific knowledge

To the best of our knowledge data about postoperative quality of life and sexual function following surgery for benign uterine diseases as hysterectomy or myomectomy is very sparse. Available literature mainly comprises small sample sizes studies, case reports and experts `opinions. Main finding from these publications are a decrease in sexual function and quality of life (1).

First more sophisticated studies regarding this question were conducted by Zobbe et collgeagues which conducted a study comparing total laparoscopic hysterectomy and supracervical hysterectomy (2). They found an improvement in postoperative quality of life and no decrease in sexual function. However they used no validated questionnaires, which make the interpretation of these findings rather difficult.

2.3 Planned observation timeline

Recruitement will start after obtaining the ethical board approval and will be conducted for the next years as part of clinical routine.

2.4 Necesity of study on humans

Not applicable

**2.4.1 Study on healthy subjects**

Not applicable

**2.4.2 Studiy on patients?**

Yes

**2.4.3 Inclusion criteria**

Included will be all patientins undergoing surgery for benign gynecological pathologies at the department of Gynecology and Obstetrics, Saarland university hospital. Patients have to provide written consent and need to be over 18 years old.

**2.4.4 Exclusion criteria**

Patients under 18 years of age and patients who do not consent.

2.4.5 Drop-out criteria

Not applicable

**3. Literature**

1) Raboch et al.: Sex life following hysterectomy. Geburtshilfe

Frauenheilkd. 1985 Jan;45(1):48-5a

2) [Zobbe V](http://www.ncbi.nlm.nih.gov/pubmed?term=Zobbe V%5BAuthor%5D&cauthor=true&cauthor_uid=14756739) et al.: [Acta Obstet Gynecol Scand.](http://www.ncbi.nlm.nih.gov/pubmed/14756739#%23) 2004 Feb;83(2):191-6. Sexuality after total vs.

subtotal hysterectomy.

**4. Explanation of advantages and meaning of study findings on patients`care**

**a) Patients`care**

By collecting more information about postoperative sexual function and quality of life following gynecological benigne procedure, preoperative counseling could be improved.

**b) Research**

Not applicable

**4.1. Participants under the age of 18 years**

Not applicable

**4.2. Individual indications**

Not applicable

**5. Balancing between disadvantages and risk by study participation and presumable advantages**

**5.1.**

No disadvantages or additional riks are caused by participation in the study.

**5.2.**

Not applicable

**5.3**

Not applicable

**6. Preoperative counseling of study participants**

**6.1**

Preoperative counseling is conducted during the preoperative work-up and in a written manner. Participation of the study is optional and written consent is taken, Return of the questionnaire is optional and in a pseudonymous manner.

**6.2 Counseling about cancellation of participation**

Patients are counseled that they can cancel their participation at any point without reasoning or disadvantages.

**6.3**

Not applicable

**6.4.**

Not applicable

**6.5.**

Not applicable

**6.6**

Not applicable

**6.7**

Not applicable

**7. Example of consignment form**

Attached

**8. Patients`insurance**

No special insurance is needed

**9. Any other requirements for realization of the study**

Not applicable

**10. Information of general practitioner**

Not applicable

**11.**

Not applicable
